# The effectiveness of group music reminiscence therapy for people thriving with dementia: A systematic review of randomized controlled trials

**DOI:** 10.1002/agm2.12344

**Published:** 2024-08-17

**Authors:** Alwin Ru Kiet Wong, Li Ting Eileen Ng, Ming Hao Lee, James Lai Hock Yeow, Yong Jia Lim, Kah Hui Yap

**Affiliations:** ^1^ School Of Applied Psychology, Social Work & Policy Universiti Utara Malaysia Kedah Malaysia; ^2^ School of Psychology, Counselling, and Family Therapy, Wheaton College Wheaton Illinois USA; ^3^ Department of Humanities and Social Sciences Nanyang Technological University Singapore Singapore; ^4^ Department of Psychology, Faculty of Behavioral Sciences HELP University Selangor Malaysia; ^5^ Faculty of Communication and Creative Industries Tunku Abdul Rahman University of Management and Technology Kuala Lumpur Malaysia; ^6^ Department of Rehabilitation, Allied Health Division Thomson Hospital Kota Damansara Selangor Malaysia

**Keywords:** behavioral and psychological symptoms, cognition, dementia, music, reminiscence therapy

## Abstract

Dementia is characterized by a progressive decline in cognition, behavioral and psychological symptoms (BPSD), and quality of life (QoL). The lack of curative therapies has led to a psychosocial discourse prioritizing QoL of people thriving with dementia (PTD). Group reminiscence therapy (RT) is a relatively inexpensive intervention, with music prompts being a preferred choice, owing to robust musical memory in the early disease stage. However, a synthesis of current evidence is needed to inform research and clinical use of group music RT in dementia care. Therefore, we conducted a systematic review on PubMed, Scopus, CINAHL, APA PsycInfo, and APA PsycArticles to critically appraise published randomized controlled trials examining group music RT to improve cognition, BPSD, and QoL in PTD. Of 14,725 articles, two RCTs involving 102 PTD were included. All studies used prerecorded music for group music RT. All studies were deemed of good quality, adhering to intention‐to‐treat analysis and assessor blinding. Based on the American Academy of Neurology guidelines, we assigned a Level C recommendation for group music RT for cognition and Level B recommendations for BPSD and QoL (ineffective). In conclusion, group music RT may be useful for symptomatic management in PTD. However, heterogeneous study designs, disease severity, dementia subtype, and outcome measures are likely barriers to meaningful clinical translation. Therefore, the rating of recommendations only serves as a point of reference. Future avenues include live performances as prompts for group music RT.

## Graphical abstract

We assigned a Level C recommendation for group music reminiscence therapy for cognition, and Level B recommendations for behavioral symptoms and quality of life (ineffective). Small number of studies, heterogeneous study designs, disease severity, dementia subtype, and outcome measures are likely barriers to meaningful clinical translation.

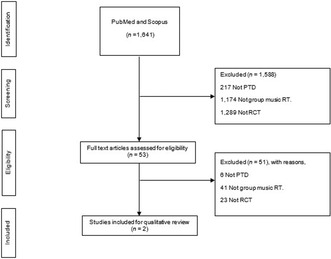

## 1 INTRODUCTION

Dementia is an umbrella term that describes an irreversible, progressive decline in cognitive function that significantly affects a person's ability to perform everyday activities.^1^ According to the International Classification of Diseases, Eleventh Revision (ICD‐11), dementia is characterized by marked impairment in two or more cognitive domains relative to that expected given the individual's age and general premorbid level of cognitive functioning.^2^ People thriving with dementia (PTD) typically experience a series of behavioral and psychological symptoms of dementia (BPSD), leading to a poor quality of life (QoL) for both the PTD and their caregivers.^3^ In view that there is no current curative therapy for dementia, there is, therefore, a need to shift toward a psychosocial discourse of dementia to improve their QoL.^4^

Reminiscence therapy (RT), relatively inexpensive, aims to provide an enriching and purposeful life for PTD using their life histories.^5^ Particularly, with the aid of tangible prompts and familiar items from the past, this is achieved by the discussion of past activities, events, and experiences with another individual or in a group.^6^ The theoretical foundation of RT, rooted in the life review model, encourages individuals to reflect on and reinterpret memories with positive significance.^7^ RT further supports the principle of preserving personhood by fostering a deeper understanding of the individual.^8^ In addition, the continuity theory suggests that revisiting an individual's past experiences can enhance memory and provide personal meaning.^9^ Collectively, these frameworks highlight the potential of RT in addressing the psychological well‐being and personal identity of PTD.

A recent meta‐analysis of 24 randomized controlled trials (RCTs) revealed that RT interventions stimulate social interaction and communication among peers,^10^ which in turn improved BPSD and QoL in PTD with small to moderate effect sizes (*d* = 0.33–0.54).^11^ It is, however, noted that in those previous studies, the use of prompts was not consistently employed, or at the very least, not explicitly reported.^11^ In those studies that reported the use of prompts, many reported the importance of it.^12^, ^13^ For example, one Japanese study used relevant activities as prompts (i.e., making and eating *onigiri*—Japanese rice balls) for group RT, suggesting that relevant prompts are essential.^12^ Similarly, a Singapore study incorporated culturally relevant art appreciation and art making into group RT with favorable results on behavioral disturbances among PTD.^13^ However, one of the challenges with most social prescribing approaches is their cost and the need for specially trained staff for their implementation.^14^ In support, the effectiveness of RT varies depending on the mode of administration and context it takes place.^6^ Therefore, exploring more alternatives may be necessary.

Music is often a preferred alternative among the prompts used in RT. Specifically, individualized music selection that is culturally relevant serves as a key feature for prompts.^15^ This preference may have stemmed from the intact musical memory despite their functional memory decline in PTD.^16^ In fact, PTD are capable of correctly perceiving pitches and melodies, recognizing familiar songs, and recalling familiar lyrics.^17^ Building on this foundation, a previous systematic review of five group music‐based interventions that used individualized music as prompts has evidenced the beneficial effects on the mental well‐being of the elderly.^18^ Additionally, music‐based interventions have also been shown to reduce cortisol and stress levels, alleviating the cognitive symptoms of PTD, which theoretically correlates with the dysregulation of the hypothalamic–pituitary–adrenal axis and increase cortisol secretion to increase cognitive impairment and BPSD in PTD.^19^

Based on our literature review, both RT and individualized music‐based interventions have demonstrated promising effects on the mental health well‐being of PTD. Existing studies shed light on the potential benefits of combining these therapies to improve the QoL for PTD and their caregivers. Although systematic reviews and meta‐analyses on RT and music‐based interventions are available, there is still a gap for an updated review focusing specifically on group music RT. Furthermore, the objective evidence regarding the efficacy of RT remains inconsistent.^5^ Nevertheless, RT represents a relatively inexpensive intervention, offering significant potential for further investigation.^5^ Therefore, we systematically examined different outcome measures, including cognition, BPSD, and QoL. We evaluated existing RCTs on group music RT for PTD. Specifically, we aimed to establish an evidence‐based guide for the group music RT in the context of PTD.

## 2 METHODS

### 2.1 Search strategy and study selection

We performed a literature search in the following electronic databases: PubMed, Scopus, CINAHL, APA PsycInfo, and APA PsycArticles. The search aimed to extract RCTs using music RT that either exclusively or predominantly involved PTD. Regarding the latter, this meant that ≥50% of patients within the study cohort had to have a dementia diagnosis. Similarly, we discarded RCTs that predominantly involved mild cognitive impairment (MCI), an intermediate stage between normal aging and dementia.^20^ The search was conducted on April 15, 2024. We used the Medical Subject Headings terms such as (1) dementia, (2) music therapy, (3) music intervention, and (4) reminiscence therapy. We included only original articles written in English.

### 2.2 Data extraction and analysis

We extracted and analyzed the data in accordance with the latest Preferred Reporting Items for Systematic Review guidelines.^21^ We used the following Population, Intervention, Comparator, Outcome, and Study design (PICOS) criteria: (1) PTD; (2) group music RT; (3) waitlist; (4) cognition, BPSD, and QoL; and (5) RCTs. The first author performed an initial eligibility screening by manually assessing the titles and abstracts of all search results, with another reviewer cross‐checking the process. Both reviewers then further assessed the full‐text versions of the eligible articles. Data on the researchers, year of publication, country, details of group music RT, sample size, clinical characteristics, clinical scales, and clinical outcomes were extracted and tabulated.

We also used validated clinical scales as outcome measures. Subscales of established clinical scales were not analyzed due to the lack of validation and their vulnerability to floor and ceiling effects, rendering them relatively insensitive when used in isolation.^22^, ^23^

### 2.3 Methodological quality

We assessed the risk of bias according to the Cochrane Collaboration's risk of bias tool,^24^ categorizing the risk of bias as high, low, or unclear in the five categories as follows: (1) randomization; (2) deviations from intended interventions; (3) missing outcome data; (4) outcome measurement; (5) selection of the reported results. Studies that lacked assessor blinding or did not adhere to intention‐to‐treat analysis were deemed to have a high risk of bias.

We then determined the levels of evidence based on the American Academy of Neurology (AAN), where studies were classified based on rating of recommendations into Level A, Level B, Level C, and Level U.^25^ Since we only included RCTs in this systematic review, we only considered Level A, B, and C recommendations. Level A suggested that a treatment that is established as effective/ineffective and requires at least two consistent Class I evidence. Level B suggested that a treatment is probably effective/ineffective and requires at least one Class I or two consistent Class II evidence. Level C suggested that a treatment is possibly effective/ineffective and requires at least one Class II evidence.^25^

## 3 RESULTS

### 3.1 Study characteristics and methodological quality

**FIGURE 1:**
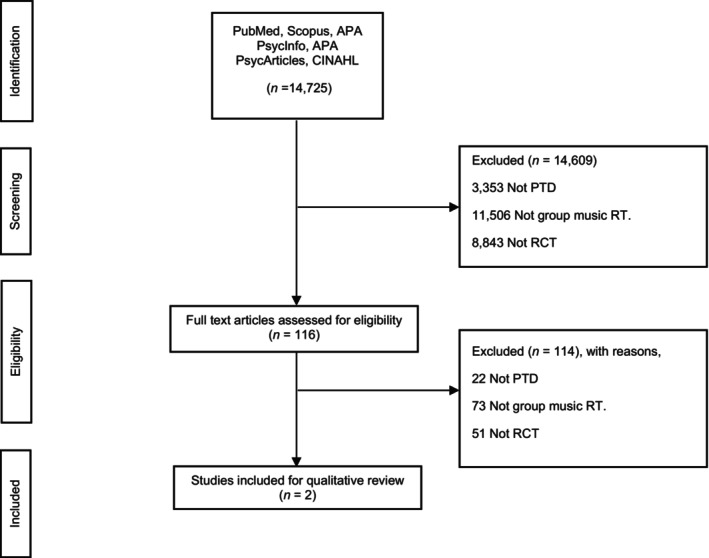
Study search per the PRISMA guideline.^21^

We screened 14,725 articles. Of these, only two studies fulfilled our inclusion and exclusion criteria (Figure 1). Both studies adhered to intention‐to‐treat analysis and assessor blinding (Table 1). One study each had Class I and Class II evidence (Table 2).

**TABLE 1:** Risk of bias assessment of the included studies based on Collaboration's risk of bias tool.

First author (year)	Risk of bias assessment				
	#1	#2	#3	#4	#5
Särkämö et al.^27^	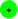	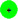	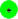	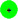	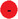
Lin et al.^26^	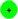	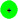	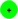	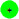	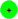

Green circle: low risk; yellow circle: unclear; red circle: high risk.

Criterion 1: Bias arising from the randomization process.

Criterion 2: Bias due to deviations from intended interventions.

Criterion 3: Bias due to missing outcome data.

Criterion 4: Bias in measurement of the outcome.

Criterion 5: Bias in selection of the reported results.

**TABLE 2:** Class of evidence of the included studies based on AAN Classification of Evidence for Therapeutic Studies.

First author (year)	Criteria for rating therapeutic studies					Class of evidence
	#a	#b	#c	#d	#e	
Särkämö et al.^27^	**✓**	**✓**	**✓**	**✓**	—*	II
Lin et al.^26^	**✓**	**✓**	**✓**	**✓**	N/A	I

Abbreviation: N/A, not applicable.

Criterion a: Concealed allocation.

Criterion b: Primary outcome(s) clearly defined.

Criterion c: Exclusion/inclusion criteria clearly defined.

Criterion d: Adequate accounting for dropouts (with at least 80% of enrolled subjects completing the study) and crossovers with numbers sufficiently low to have minimal potential for bias.

Criterion e: *Only required for noninferiority or equivalence trials claiming to prove efficacy for one or both drugs.

Among the included studies, a double‐blind RCT (Class I) examined the effect of a 60‐min, twice‐a‐week, and over a period of 4 weeks, Taiwan festival‐themed group music RT incorporating relevant music, pictures, and somatosensory interactive games delivered through virtual reality (VR) (*n* = 25), and compared them to the control group (*n* = 20).^26^ A single‐blind RCT (Class II) examined the effect of a 90‐min, once‐a‐week over a period of 10 weeks, personalized group music RT with album covers as visual cues (*n* = 29) and compared them to the control group (*n* = 28).^27^ Both studies used prerecorded music as a prompt for RT (Table 3, details in Table S1).

**TABLE 3:** Outcome measures of the included studies.

First author (year)	Cognition					BPSD			QoL	
	General	Abstract reasoning	Executive function/Attention	Language	Memory	General	Depression	Anxiety	PTD	Caregivers
^27^ ^a^	MMSE	Similarities Block design Sequential commands	FAB Digit span Trail making test	Verbal fluency Boston naming test	Logical memory Word list				CBS QoL‐AD	GHQ ZBI
^26^	SPMSQ Intervention: Baseline: 6.400 ± 2.300 4‐weeks: 6.000 ± 2.500 Control: Baseline: 4.600 ± 3.000 4‐weeks: 5.100 ± 3.000					NPIQ (Severity) Intervention: Baseline: 7.800 ± 6.200 4‐weeks: 8.200 ± 6.200 Control: Baseline: 9.400 ± 8.100 4‐weeks: 8.400 ± 8.000	CSDD Intervention: Baseline: 5.600 ± 4.700 4‐weeks: 5.200 ± 4.600 Control: Baseline: 6.800 ± 6.600 4‐weeks: 11.700 ± 7.200			NPIQ (Caregiver Stress) Intervention: Baseline: 8.200 ± 7.100 4‐weeks: 8.800 ± 6.900 Control: Baseline: 12.400 ± 11.700 4‐weeks: 9.700 ± 10.300

Abbreviations: BPSD, behavioral and psychological symptoms of dementia; CBS, Cornell‐Brown Scale for Quality of Life in Dementia; CSDD, Cornell Scale for Depression in Dementia; FAB, Frontal Assessment Battery; GHQ, General Health Questionnaire; MMSE, Mini Mental State Examination; NPIQ, Neuropsychiatric Inventory Questionnaire; PTD, Patients thriving with dementia; QoL, Quality of life; QoL‐AD, Quality of Life in Alzheimer's Disease; SPMSQ, Short Portable Mental Status Questionnaire; ZBI, Zarit Burden Interview.

^a^ Means and SD not reported.

There were a total of 102 participants in the included studies. Both studies used the Clinical Dementia Rating (CDR) as a diagnostic criterion, with participants' severity ranging from 0.5 to 2, with 0.5, 1, and 2 corresponding to MCI, mild, and moderate dementia.^28^ Neither study reported the number of PTD in terms of disease severity (Alzheimer's: *n* = 28, Vascular: *n* = 13, mixed: *n* = 7, and others/unspecified: *n* = 54). Outcome measures were categorized into changes in cognition, BPSD, and QoL (Table S1).

### 3.2 Cognition

Class I: A double‐blind RCT of Taiwanese festival‐themed group music RT did not improve general cognition in PTD over 4 weeks using the Short Portable Mental Status Questionnaire (SPMSQ), compared to the control group.^26^

Class II: A single‐blind RCT of personalized group music RT improved general cognition, executive function/attention, and memory in PTD. However, these improvements became nonsignificant after post hoc analysis.^27^

### 3.3 Behavioral and psychological symptoms of dementia

Class I: Double‐blind RCT of Taiwanese festival‐themed group music RT did not improve the severity of neuropsychiatric symptoms in PTD over 4 weeks as assessed by the Neuropsychiatric Inventory‐Questionnaire (NPIQ) compared to the control group. However, improvement was shown in depressive symptoms using the Cornell Scale for Depression in Dementia (CSDD).^26^

### 3.4 Quality of life

Both Taiwanese festival‐themed group music RT (Class I) and personalized group music RT (Class II) did not improve the QoL of PTD and caregivers compared to the control group.^26^, ^27^

## 4 DISCUSSION

In this review, we systematically evaluated and summarized two RCTs of group music RT for PTD. We rated the level of recommendations for cognition, BPSD, and QoL based on the AAN guidelines,^25^ and lastly, given that the studies employed heterogeneous outcome measures, we did not compare the effects across types of group music RT.

We assigned a Level C recommendation to group music RT as possibly effective in improving cognition in PTD based on one Class II study.^27^ While a post hoc analysis minimizes the risk of Type I error, some true positives may not survive the alpha‐level corrections.^29^ Additionally, this study recruited PTD with severity ranging from MCI to moderate dementia.^27^ It is possible that MCI has not exceeded the threshold for irreversible damage, and the remaining brain cells are able to respond to potential treatment for cortical reorganization. In contrast, neuronal loss in mild and moderate dementia may lead to the absence of target cells to respond to the treatment,^30^ thereby masking the potential improvement in MCI. Furthermore, MCI would have easily exceeded the ceiling effect with sufficient neuroplasticity,^31^ which may explain the nonsignificant improvement trend.

There are two possible reasons that may explain the heterogeneous findings between the two studies on cognitive function. Firstly, screening tools like SPMSQ are susceptible to ceiling effects and insensitive to change.^26^ While cognitive screening tools are useful in monitoring cognitive decline in dementia,^31^ a standardized full neuropsychological assessment is more sensitive in assessing the range of cognitive domains.^23^ Secondly, the differences in duration (i.e., 4 weeks vs 10 weeks) may also help to explain the differences in outcome; that is, a minimum duration of group music RT is needed for cortical reorganization to take place and manifest into clinical outcomes.

Overall, the findings from this review align partially with a recent meta‐analysis that reported RT has a beneficial effect on cognition in PTD.^32^ Therefore, future studies should focus on specific severity groups and use individual cognitive tests.

Although group music RT may have received a Level B recommendation for the probable ineffectiveness in managing BPSD, it is evidenced as effective in improving depressive symptoms (Level B).^26^ Such findings are likely attributed to the nature of the measure, the NPIQ.^33^ The NPIQ assesses the severity of neuropsychiatric symptoms and caregiver distress using a short range of scores, lacking the details and breadth of symptoms, which likely interferes with the lack of sensitivity to change.^34^ Therefore, future trials should investigate changes in individual BPSD severity in isolation, (e.g., depression via CSSD,^26^ agitation via the Cohen‐Mansfield Agitation Inventory Observational tool).^35^

Finally, we assigned a Level B recommendation to group music RT for the probable ineffectiveness in improving QoL.^26^, ^27^ There is no conceptual consensus on what constitutes health‐related QoL, as reflected in heterogeneous measures used in the studies, and we adopted a multidimensional model, including physical, cognitive, and activities of daily living aspects to conceptualize QoL in this study.^36^ Future studies could benefit from adopting a more standardized protocol in objective measures for QoL.

### 4.1 Implications

This review highlights several research gaps in the literature. First, group music RT has not been comprehensively evaluated in PTD, with only two RCTs, and a Level B recommendation at best. Level C recommendation in cognition was given as it aligns with the core impairment in dementia, due to insufficient neuroplasticity in the brain regions implicated in the cognitive function of PTD to respond to the treatment.^31^ This is helpful in reducing the duplication of group music RT research targeting cognition and leads to a more prudent utilization of resources for BPSD.

Second, both studies investigated group music RT that used recorded music as their prompts. While recorded music offers more consistent effects on behavioral disturbances, live music offers additional benefits on interpersonal connections and social relationships, providing a greater interactive feature and more meaningful participation.^37^ However, it is also worth noting that the inconsistency in live music may be due to individual differences in the communicative or interactional processes of active participation. In addition, musicians' level of training (i.e., professional therapist vs professional performer vs amateur), including the quality of performance and experience conducting the RT, may also contribute to the efficacy of the group music RT.

Third, aligning with the importance of the continuity theory for RT,^9^ both studies used culturally relevant music. However, given that RT emphasizes on the preservation of the personhood,^8^ personal preference for the genre of music (e.g., pop, jazz, and classical) likely played a part in the facilitation of group music RT compared to the relevance of the music.^38^ Therefore, the effectiveness of group music RT may require tailoring personal choice to the music selections of the participants (i.e., generation, ethnicity, culture, and language), which may result in a hefty cost.^14^

Fourth, we specified group music RT as part of the inclusion criteria. A systematic review reported that group RT was more effective as compared to individual sessions in improving psychological factors in the elderly.^39^ The implementation of group RT stimulates social interaction and communication among peers, which has beneficial effects on QoL.^10^ Similar to music selection, it is also important to consider the individual characteristics of the participants during group music RT.

The absence of adverse events suggested that the mode of administration (e.g., VR) and duration are tolerable for PTD in these studies (i.e., at least 60‐min twice weekly sessions). This finding may imply that future studies could consider VR‐based interventions for PTD. In fact, a recent systematic review has shown that VR was well tolerated by PTD with high engagement.^40^ Nevertheless, the individual prevalence of VR sickness still needs to be assessed before introducing such modality.^41^ On a similar note, fatigue should also be monitored to tailor the treatment duration accordingly.^42^

### 4.2 Limitations

This review has several limitations. Firstly, heterogeneous study designs (e.g., mode of administration), additional prompts (e.g., album cover), elements, (e.g., somatosensory interactive games), duration, and outcome measures complicated comparisons across studies. The latter was partially affected by conceptual differences in cognitive domains and QoL.^23^, ^36^ Second, heterogeneous inclusion criteria led to uncertainty regarding whether these findings could generalize to PTD of varying severity. Third, different dementia diagnoses were included, with 54 out of 102 were unspecified. Comparing treatment effects across different subtypes and identifying diagnosis‐specific effects is impossible. Fourth, we only included RCTs and may have missed other group music RTs that could be relevant. Additionally, the small number of RCTs, together with heterogeneous study design, rendered meta‐analysis impossible. Lastly, despite being as comprehensive as possible in the MeSH search terms, our search may have missed some articles due to the selection of alternative keywords.

### 4.3 Future directions and conclusion

In conclusion, this review highlights the lack of RCTs examining the effect of group music RT on PTD. Group music RT received Level C recommendation for cognition, Level B recommendation for depression, and an ineffective Level B recommendation for QoL. However, both studies were heterogeneous, and the ratings should only serve as a reference. These studies could benefit from investigating the effectiveness of group music RT on the various stages of dementia severity, specific dementia diagnoses, and the selection of appropriate outcome measures (i.e., cognitive and BPSD tests that measure individual domains, a multidimensional QoL questionnaire). Future RCTs should also investigate the effect of group music RT in the form of live performances, which could offer alternatives for PTD and help develop new social prescribing. Since existing meta‐analyses were based on music‐based intervention in general, more studies will allow a robust rating of recommendation and meta‐analysis of group music RT.

## CONFLICT OF INTEREST STATEMENT

We declare that there is no existing conflict of interest between any of the researchers in the study and their respective organizations.

## Supporting information


Table S1.

## Data Availability

Lastly, the data supporting this study's findings are available in this article.
