# Supplementary material for: The effectiveness of group music reminiscence therapy for people thriving with dementia: A systematic review of randomized controlled trials
Source: Aging Med (Milton). 2024 Aug 17;7(4):528–34. doi: 10.1002/agm2.12344 (PMC11369345; doi:10.1002/agm2.12344)
Supplement: Supplementary file 1 — Table S1. [file AGM2-7-528-s001.docx]

Supplementary Table 1.

*Characteristics of RCTs for group music RT in PTD.*

| First author (year)  Country | Study design and Class of evidence | Intervention(s), Control and setting [Group music RT schedule] | Sample size | Main clinical characteristics | Outcome measure(s) | Clinical outcome(s) [Mean (SD)] | Findings |
| --- | --- | --- | --- | --- | --- | --- | --- |
| (Särkämö et al., 2014)  Finland | Single-blind RCT  Class II | Intervention:  Personalized (along with visual cues)  [90 min, once a week, over 10 weeks]  Control:  Usual care | **Intervention:**  29  **Control:**  28 | **Mean age (years)**  Intervention:  79.4 ± 10.1  Control:  78.4 ± 11.6    **Gender [M/F]**  Intervention:  3/26  Control:  10/18  **Education level (years)**  Intervention:  2.8 ± 2.0  Control:  3.0 ± 1.7  **Dementia type**  Intervention:  14 AD, 7 VaD, 4 Mixed, 4 Others  Control:  14 AD, 6 VaD, 3 Mixed, 5 Others  **Diagnostic criteria**  CDR: 0.5 – 2 | **Cognition**  MMSE  FAB  WMS III   - Digit Span Backward - Logical Memory I - Logical Memory II   CERAD   - Word-list Learning - Word-list Delayed Recall - Verbal Fluency   WAIS III   - Similarities - Block Design   Boston Naming Test  Western Aphasia Battery   - Sequential Commands   Trail Making Test Part A  **QoL**  CBS  QoL-AD   - Self-report - Caregiver-report   ***Caregiver**  **QoL**  GHQ  ZBI | *Outcomes not reported individually.  *Post-intervention outcomes not reported. | Intervention improved mood, orientation, and remote episodic memory and to a lesser extent, also attention and executive function and general cognition; as well as QoL, compared to control. |
| (Lin, Wu, Chen, & Huang, 2023)  Taiwan | Double-blind RCT  Class I | Intervention:  Taiwanese festival-themed  [60 min, twice a week, over 4 weeks]  Control:  Usual care | **Intervention:**  25  **Control:**  20 | **Mean age (years)**  Intervention:  83.9 ± 7.7  Control:  81.4 ± 7.9    **Gender [M/F]**  Intervention:  4/21  Control:  5/15  **Education level [Elementary and below/Junior and above]**  Intervention:  22/3  Control:  13/7  **Dementia type**  Unspecified dementia  **Diagnostic criteria**  CDR: 0.5 – 1 | **Cognition**  SPMSQ  **BPSD**  CSDD  NPIQ | SPMSQ  Intervention:   - Baseline: 6.4 ± 2.3 - 4-weeks: 6.0 ± 2.5   Control:   - Baseline: 4.6 ± 3.0 - 4-weeks: 5.1 ± 3.0   NPIQ (Severity)  Intervention:   - Baseline: 7.8 ± 6.2 - 4-weeks: 8.2 ± 6.2   Control:   - Baseline: 9.4 ± 8.1 - 4-weeks: 8.4 ± 8.0   NPIQ (Caregiver Stress)  Intervention:   - Baseline: 8.2 ± 7.1 - 4-weeks: 8.8 ± 6.9   Control:   - Baseline: 12.4 ± 11.7 - 4-weeks: 9.7 ± 10.3   CSDD  Intervention:   - Baseline: 5.6 ± 4.7 - 4-weeks: 5.2 ± 4.6   Control:   - Baseline: 6.8 ± 6.6 - 4-weeks: 11.7 ± 7.2 | Intervention non-significantly improved cognition and BPSD compared to control. |

Abbreviations: BPSD, behavioral and psychological symptoms of dementia; CBS, Cornell-Brown Scale for Quality of Life in Dementia; CDR, Clinical dementia rating; CERAD, Consortium to Establish a Registry for Alzheimer’s Disease battery; CSDD, Cornell Scale for Depression in Dementia; FAB, Frontal Assessment Battery; MMSE, Mini Mental State Examination; NPIQ, Neuropsychiatric Inventory Questionnaire; QoL, Quality of life; QoL-AD, Quality of Life in Alzheimer’s Disease; SPMSQ, Short Portable Mental Status Questionnaire; WAIS, Wechsler Adult Intelligence Scale; WMS, Wechsler Memory Scale; ZBI, Zarit Burden Interview.

Lin, T.-H., Wu, W.-R., Chen, I.-H., & Huang, H.-C. (2023). Reminiscence music intervention on cognitive, depressive, and behavioral symptoms in older adults with dementia. *Geriatric Nursing*, *49*, 127-132.

Särkämö, T., Tervaniemi, M., Laitinen, S., Numminen, A., Kurki, M., Johnson, J. K., & Rantanen, P. (2014). Cognitive, emotional, and social benefits of regular musical activities in early dementia: Randomized controlled study. *The Gerontologist*, *54*(4), 634-650.
